# The absorption and multiplication of uncertainty in machine‐learning‐driven finance

**DOI:** 10.1111/1468-4446.12880

**Published:** 2021-07-27

**Authors:** Kristian Bondo Hansen, Christian Borch

**Affiliations:** ^1^ Department of Management, Society and Communication Copenhagen Business School Frederiksberg Denmark; ^2^ Department of Management, Politics and Philosophy Copenhagen Business School Frederiksberg Denmark

**Keywords:** algorithms, economic sociology, financial models, machine learning, uncertainty

## Abstract

Uncertainty about market developments and their implications characterize financial markets. Increasingly, machine learning is deployed as a tool to absorb this uncertainty and transform it into manageable risk. This article analyses machine‐learning‐based uncertainty absorption in financial markets by drawing on 182 interviews in the finance industry, including 45 interviews with informants who were actively applying machine‐learning techniques to investment management, trading, or risk management problems. We argue that while machine‐learning models are deployed to absorb financial uncertainty, they also introduce a new and more profound type of uncertainty, which we call critical model uncertainty. Critical model uncertainty refers to the inability to explain how and why the machine‐learning models (particularly neural networks) arrive at their predictions and decisions—their uncertainty‐absorbing accomplishments. We suggest that the dialectical relation between machine‐learning models’ uncertainty absorption and multiplication calls for further research in the field of finance and beyond.

## 1 INTRODUCTION

Being able to transform impalpable economic uncertainties into manageable risk is a fantasy of many financiers. The sophistication and capacity of predictive tools such as big‐data processing machine‐learning (ML) algorithms (Mackenzie, 2015a) make treating uncertainty as a “resource” (Esposito, 2013) rather than a threat or obstacle appear attainable. Consequently, a significant inflow of new data‐mining and data‐processing technologies are profoundly changing investment management and securities trading (Guida, 2019; Lopez de Prado, 2018). Banks, hedge funds, trading firms, and clearing firms increasingly adopt ML techniques not merely to maximize information from their data, but fundamentally, to tame uncertainty.

The ambition of reducing uncertainty through ML techniques is empirically and theoretically significant. Empirically, it enlarges a decades‐long development where computers and algorithms gradually replace humans in key functions within financial markets (Borch et al., 2015). Where human traders previously dealt with uncertainty, this task is increasingly delegated to machines. Enhanced data availability, computing power, reduced data storage costs, and the automated market infrastructures have accelerated this development (Arnott et al., 2018; Kirilenko & Lo, 2013; Pardo‐Guerra, 2019). The use of ML is augmented by trading firms’ belief that this technology will enable sufficient “edge” vis‐à‐vis competitors, that is, provide them with predictive powers to make the *future* (and future price movements) less uncertain. Elsewhere in finance, ML is getting traction because an explosion of market data has necessitated new approaches to comprehend a *past* that appears increasingly uncertain. With the rise of automated trading (MacKenzie, 2018), the number of orders to buy or sell financial assets has risen dramatically. Mattli (2019, p. 142) reports that in the USA, the Financial Industry Regulatory Authority “monitors on average about 50 billion market events (quotes, cancellations, and trades) a day across equities, options, and a few other markets.” This explosion of data makes establishing past events (even over the past trading day), including any market misconduct, increasingly uncertain, and ML is a means to absorb such uncertainty. To be sure, it is not uncommon to see conflicting interpretations about past events also in pre‐ML settings, especially when these concern financial crises. What is new is that the data surge has intensified the problem of establishing what happened in the immediate past, including in mundane, non‐crisis‐driven situations.

Theoretically, the inflation of uncertainty and ML‐based solutions prompts a re‐evaluation of discussions in economic sociology that often revolve around Knight’s (1921) famous distinction between measurable risk and immeasurable uncertainty—hence “Knightian uncertainty” (Basili, 2001; Svetlova, 2012). Drawing on Knight's understanding of true uncertainty and, responding to Beck’s (2009, pp. 17–18) apparent confusion of risk and uncertainty, Pixley (2014, pp. 201–202) argues that “the financial future is not certain ‘in parts,’ it is unavoidably unknowable.” To compartmentalize the future into knowable probabilities is, according to Pixley, an illusion. Conversely, Power (2004, p. 53) labels Knight's distinction as “historical and changing rather than invariant” and contends that “incalculable uncertainties can be ‘tamed’ as calculable risks.” Similarly, Carruthers (2013, p. 528) demonstrates that credit rating agencies have fostered a “domestication” of credit uncertainties, which means moving uncertainty toward risk. However, he stresses that the transformation of uncertainties into risks is “never entirely complete or successful” (p. 527).

This article contributes to this discussion by analyzing uncertainty in ML‐driven finance. Interviews with practitioners who utilize financial ML models informed our analysis to concur with Power that Knight's distinction is historically variant. The domain of finance contemplates an uncertain future that needs taming and an increasingly uncertain past and present. We argue that while ML models are able to absorb or domesticate uncertainty, they also introduce new, sometimes more profound, uncertainties. Particularly as the inner workings of sophisticated ML systems are often highly opaque, “financial uncertainty” (about past and future financial events) is replaced by what Varian (2014, p. 24) calls “model uncertainty,” meaning uncertainty about how ML models reduce (financial) uncertainty. Beckert (2016, p. 45) suggests that uncertainty is fundamental to decision‐making because “either actors do not fully understand all the parameters relevant to a situation's outcome because of complexity, or all of the relevant factors do not yet exist.” We argue that ML algorithms in financial markets sometimes render the future more tangible and predictable but simultaneously introduce model‐endogenous uncertainty.

This article demonstrates the uncertainty‐absorbing properties and uncertainty‐inducing side effects of ML and how this dialectic between the simultaneous reduction and production of uncertainty affects the application of these techniques to financial problems. Thus, the article contributes to the literature on uncertainty in economic sociology and the social studies of finance. Several economic sociologists have explored uncertainty (Arnoldi, 2006; Beckert & Berghoff, 2013; Carruthers, 2013; Chong & Tuckett, 2015; Esposito, 2013; Esposito & Stark, 2019; van der Heide, 2020; Langley, 2012; Lockwood, 2015; Pixley, 2014). However, this article is, to our knowledge, the first to explore how market participants deal with and are affected by the uncertainty associated with the use of ML models in financial markets. Nevertheless, given its focus on the dialectical relation between the uncertainty ML models absorb and produce, we believe our analysis has salience as a lens to examine additional fields where ML is an uncertainty‐absorbing technology.

After this introduction follows a section that examines uncertainty in model‐mediated decision‐making in financial markets. We argue that uncertainty assumes two forms, one concerning the past and the future, termed *natural uncertainty*, and another about the operations of ML models, titled *critical model uncertainty* (Thévenot, 2002). Subsequently, we present a methods and data sources section. In sections four and five, we examine how ML models absorb past and future financial uncertainty while producing model uncertainty and discuss what critical model uncertainty means for model‐driven finance and market participants. The final section concludes the article.

## 2 UNCERTAINTY AND MODEL‐MEDIATED DECISION‐MAKING

As practitioners recognize that financial models handle uncertainty imperfectly, they use them with cautious human judgment, the reason for which is captured in Svetlova’s (2013) study of financial valuation models that discusses the variables these models depend on:

[T]he determinant variables of valuation models (future cash flows and the discount rate) depend on other factors, including various macro‐parameters, characteristics of the political situation, specific company developments, and market sentiment. These variables can be divided into further refined variables (e.g. oil prices, core inflation, tax policy and others) that are specified neither in the model nor elsewhere. These refined variables are knowable from experience, and each investor measures their importance differently. Second, because those factors consist mostly of expectations instead of observable data, they always interact with each other. Furthermore, expectations have a recursive and reflexive character: in social systems, such as financial markets, expectations influence the realization of phenomena and, in turn, are influenced by realizations. Because of this reflexivity, most factors that are relevant for valuations are not independent and not stable. (Svetlova, 2013, p. 323)

The recursive and reflexive character of financial markets and its effects on uncertainty is also expressed by Esposito (2011, p. 28): “The uncertainty of the future is multiplied by the uncertainty of the behaviour of all other operators who are oriented to the same future, further increasing the complexity” (see also Cevolini & Esposito, 2020). Although this diminishes hopes to absorb uncertainty, Esposito (2011, p. 28) argues that, paradoxically, the multiplication of uncertainty increases complexity and enables the structuring of uncertainty: “uncertainty appears less uncertain if others handle it in the same way.” Hence, uncertainty becomes tangible and manageable if practices align or are standardized. In finance, market participants try to reduce uncertainty through “cognitive standardization” (Preda, 2005; see also Beunza & Garud, 2007; Chong & Tuckett, 2015). Cognitive standardization is achieved with tools “with the help of which heterogeneous pieces of information are made comparable, interpreted and used in financial decisions” (Preda, 2005, p. 453). Preda lists stock‐analysis techniques, pricing formulas, and financial models as tools which “concomitantly disseminate and standardize information” (454). ML techniques can be added to this list.

Model‐ and technology‐aided cognitive standardization is a form of “uncertainty absorption” (Luhmann, 2002, 2018; March & Simon, 1993) whereby March and Simon (1993, p. 186) describe organizational situations where “inferences are drawn from a body of evidence and the inferences, instead of the evidence itself, are then communicated” to the organization's members. The example they offer is that of transforming comprehensive questionnaire data into easily accessible statistical tables and communicating them through the latter. The type of uncertainty at stake here is obviously different from Knight's conception of term. While his notion points to inherent unknowability, March and Simon's concept of uncertainty absorption does not imply that the “body of evidence” is in principle unknowable but simply that it is more practical to convey its complexity in an at once condensed and transformed form. This is an analytically valuable conceptualization because it captures how uncertainty absorption produces new uncertainty and the need for confidence in the steps taken to achieve the uncertainty absorption:

Through the process of uncertainty absorption, the recipient of a communication is severely limited in his [*sic*] ability to judge its correctness. Although there may be various tests of apparent validity, internal consistency, and consistency with other communications, the recipient must, by and large, repose his confidence in the editing process that has taken place, and, if he accepts the communication at all, accept it pretty much as it stands. To the extent that he can interpret it, his interpretation must be based primarily on his confidence in the source and his knowledge of the biases to which the source is subject, rather than on a direct examination of the evidence. (March & Simon, 1993: 186–187)

Hence, the recipient of the communication may know little about the processes and means of the uncertainty absorption. Absent insights into the original evidence, the recipient would therefore have little choice but to place confidence in the uncertainty‐absorbing process.

Elaborating on March and Simon's account, Luhmann (2018, p. 151) similarly argues that “the absorption of uncertainty […] can also increase uncertainty.” For Luhmann, this is so because uncertainty is absorbed through decisions that, given that each “include the prospect of future decisions,” generates novel uncertainties (p. 149). We argue that this dialectic between uncertainty absorption and production is particularly significant for firms deploying ML models. As more decision‐making is delegated to these poorly understood models, the uncertainties they absorb on one level are followed by increases in uncertainty on another level.

However, the types of uncertainties absorbed and produced are not the same. To theorize their difference in market settings, we utilize Thévenot’s (2002) distinction between natural and critical uncertainty as developed in the context of (commodities) markets. Natural uncertainties can be absorbed through market coordination (what Thévenot refers to as the “market principle”) and standardization:

For instance, if the uncertainty about others’ actions results from their purchasing desires, then the market principle makes it possible to overcome this natural uncertainty, thanks to the common identification of goods and to the common recognition of their prices. (Thévenot, 2002, p. 189)

Managing and mitigating natural uncertainty, therefore, absorbs uncertainty. However, market participants may face contingencies not easily domesticated through the market principle but which stand out as noise instead. Despite attempts to apply the market principle to contingencies, absorbing “exterior contingencies” (say, considering the environmental externalities of production), is bound to be imperfect (Thévenot, 2002, p. 190). Hence, uncertainty absorption has limits; something remains contingent, despite efforts to domesticate it.

While natural uncertainty is partially absorbed through market‐based coordination, classification, and standardization, “critical uncertainty” challenges the viability of uncertainty absorption. “In the case of the market world, a critical uncertainty is that which debilitates the common identification of commodities, their market objectivity.” Indeed, critical uncertainty cannot be absorbed through the market principle, or “rejected as part of the noise of contingencies”; rather it “casts doubt on the very nature of the action” (Thévenot, 2002, p. 190). Thévenot's example is of a blood donor whose decision to donate may neither be compatible with a market‐based principle (say, treating blood as a commodity) nor reducible to an act of sympathy. Disparate interpretations exist simultaneously so that “in critical situations, actors—and researchers who interpret their actions—are torn between incommensurable rationales” (Thévenot, 2002, pp. 181–182). There is no widely agreed‐upon way to understand the action and no common approach to absorb its uncertainty.

As we shall demonstrate below, financial ML models sift large unstructured, noisy datasets to extract complex structures and generalizable patterns, undefined in advance, and concerning others’ actions, thus absorbing natural uncertainty by detecting correlations in data and turning the natural uncertainty and noise into manageable, actionable risk. However, while ML helps mitigate natural uncertainty in markets, it also contributes to the creation of critical uncertainty through its complex and often indecipherable operations. Indeed, the inner workings and decision‐making logic of some models, particularly deep neural networks, are profoundly opaque, and ML experts cannot fully explain how these systems predict and decide (Burrell, 2016; Samek et al., 2019). As a result, we argue that such ML systems produce critical uncertainty, not just in the Thévenot sense of interpretations of their action playing out between incommensurable rationales, but in the more fundamental sense of rendering interpretable rationales unviable or altogether unattainable. In other words, the natural market uncertainty ML systems absorb comes at a price, namely critical model uncertainty.

Our notion of critical model uncertainty extends Thévenot's work. We further suggest that sociological discussions of risk and uncertainty be extended along a temporal dimension when it comes to ML‐based finance. Unsurprisingly, most sociological debates about uncertainty concern the future (Beckert, 2016; Esposito, 2011, 2013; van der Heide, 2020). This is understandable, but as mentioned earlier, the data explosion within financial markets has made the past and the present increasingly uncertain as well: although both are, in principle, knowable, it has become more and more difficult to establish what happened on the financial exchanges during the past trading day. To illustrate, despite massive efforts, the USA’s financial regulators’ account of the Flash Crash in May 2010, when automated trading algorithms triggered their “first generalized crisis” (MacKenzie, 2015b, p. 648), remains contested. There is no consensus about the event, the most significant parts of which lasted less than an hour (Borch, 2016). Accordingly, the uncertainty previously reserved for the future now increasingly extends into the past—even the immediate past needs to be transformed into manageable risk for market participants.

## 3 METHODS AND DATA SOURCES

ML is as complex a field as algorithmic securities trading is secretive, which poses several challenges for researchers studying the former's application in the latter. Although the secrecy of the algorithmic trading and model‐driven investment management industries have been exaggerated (Lewis, 2014; Zuckerman, 2019; cf. MacKenzie, 2017), it is difficult to access details on active trading algorithms. The source code, detailing the sequence of instructions a trading algorithm performs when executing orders, constitutes the “secret sauce” that firms fiercely guard and academics cannot access (MacKenzie, 2018, p. 1637). The fact that ML is a rather broad moniker and most useful ML systems are tailored to their context of application and comprise several different elements does not make things less complicated (Weigand, 2019, p. 85). Lowrie (2017, p. 4) describes ML as a “diverse congeries of algorithmic approaches, software implementations of such approaches, and hardware configurations designed to handle such implementations” effectively capturing the intricacies of such technical assemblages. In contrast to earlier forms of algorithmic trading where a team of human traders and software developers would translate the traders’ strategies into code, thereby defining the algorithmic rules end‐to‐end, the idea of ML is to let the system itself develop its own rules. This includes designing the system to automatically adjust its parameters to increase its predictive power, without humans intervening in this process.

Different approaches have been proposed for the ethnographic study of algorithms and their use in professional contexts. Although most scholars agree that “algorithm” is not an essentialist but a context‐dependent term, there is no consensus whether it is an emic term of technical professional cultures (Dourish, 2016), a black box “object of ignorance” (Lange et al., 2019) or a cultural artefact of those developing, using, and studying them (Seaver, 2017). These views suggest the difficultly of examining these technical devices and their impact in practice. We propose a simple approach to study the use of ML algorithms in finance, which considers the context of algorithmic conception, formulation, and application (Lenglet, 2011; Seyfert, 2016), while acknowledging the limited access to the details of the algorithms’ configurations. Focusing on the forms of uncertainty pertaining to ML algorithms, we concentrate on (1) the basic logic and operations of specific ML implementations and (2) the ways practices adapt to these technical instruments. Aligned with Christin’s (2020, p. 906) idea of “algorithmic refraction,” our approach entails “paying close attention to the changes that take place whenever algorithmic systems unfold in existing social contexts—when they are built, when they diffuse, and when they are used.”^1^

Specifically, we utilize interviews with 182 market participants working in hedge funds, proprietary trading firms, brokers, banks, exchanges, tech‐, analytics and data vendors, and regulatory bodies (Table A1—Appendix) as well as structured and more ad‐hoc observation studies in primarily quantitative hedge funds and proprietary algorithmic trading firms. The fieldwork was conducted from late 2017 to late 2020 by an interdisciplinary team studying the extensive, increasing algorithm use in the financial industry.^2^ Of the 182 semi‐structured interviews, we selected 45 interviews with market participants who were applying or had implemented ML techniques to investment management, trading, or risk management problems. We do not focus on a specific area of algorithmic finance (such as high‐frequency trading) but examine ML in algorithmic and model‐driven investment management and trading writ large. That said, most interviewed ML specialists work in trading firms, investment management (mostly, hedge funds), and brokerages (Table A2—Appendix). In addition to interview data, we draw on our observation data and written material (reports, presentations, practitioners’ articles, blog posts, etc.) about ML and artificial intelligence in finance.

When analyzing the interview transcripts, we compile instances of ML techniques and strategies in relation to specific investment, trading, and risk management approaches. We use an open coding approach to our analysis of the interviews to focus on uncertainty and applications of ML to financial problems. Our codes include noise, complexity, ignorance, comprehensibility, risk, and uncertainty. The following analytical section is about the absorption of natural uncertainty through ML techniques in risk management. Specifically, it examines the domestication of uncertainty using an ML model developed by a small quant risk team in the clearing division of a large European bank.

## 4 ABSORBING NATURAL UNCERTAINTY

The main job of the clearing bank we examined is to reconcile derivative transactions between buyers and sellers in the market. In addition to settling transactions, it finances its clients’ positions. To offset the risk of financing clients’ trading, the clearer asks clients to provide collateral, which remains in clients’ margin accounts with the bank. The clearer does *margining*—calculating what clients borrowed for trades and how much collateral they are supposed to have with the bank—daily. The collateral is a fraction of what clients borrow. If a client incurs a loss, the quant team requests more collateral.

From the bank's quant risk team, we interviewed the head and the team's sole ML specialist (K009 in Table A2—Appendix) who used various statistical models to calculate clients’ collateral.^3^ A primary task is to calculate the “worst‐case potential loss of a client's portfolio from today to tomorrow,” the team's head revealed. To this end, the team used a variation of a classic, the Value‐at‐Risk (VaR) model. Although “not strictly speaking a Value‐at‐Risk model,” the team's head emphasized, the model's objective was similar: to calculate how much a portfolio risks diminishing in value over a given period with a given probability and under normal market conditions. The cue here is that traditional statistical risk management models like the VaR make several assumptions about the markets actors involved, considering market conditions to be stable and normal (Lockwood, 2015). To bolster risk management practices and capture some uncertainty escaping the standard models, the quant team adopted ML.

The model they selected was an unsupervised artificial neural network designed to detect anomalies in financial transaction data. An artificial neural network is a computational system loosely built in the image of the human brain consisting of several nodes or artificial neurons that transmit signals through artificial equivalents of synapses. The transmission process is contingent on weights that may adapt as the system learns from processing data (Alpaydin, 2016, pp. 86–88). For the present analysis, as the quant risk team's ML specialist mentioned, “we wanted to have an approach that was unsupervised learning and that could find anomalies, that is, things that are very different from the rest of the data.”

A major feat of the bank's ML model is that it discovers irregular patterns or anomalous structures in the data, which would otherwise remain undetected. Devising such a model requires ample time, expertise and, closer to production, computational power and data storage capacity. However, before the team faced processual challenges—procedural and organizational issues of implementing a large‐scale technical innovation in a somewhat rigid organization as a big bank—the idea had to be conceived. The ML anomaly detection model emerged from a simple inference by the head of the quant risk team's observation of how seemingly underutilized data were piling up in the bank.

I observed that we had more and more data. I think four years ago [around 2014], within the clearing they started to set up a data lake and in it they started to collect and store a lot of transaction data. I thought if we store the data, we could also do something with the data and benefit from that. I then had the idea to analyse the data and try to detect anomalies. [...] Here [at the local exchange] we have four million transactions a day and around the globe eight to ten million daily transactions.

His problem, he confessed, was that he had little prior knowledge about anomaly detecting ML algorithms. He knew of ML and some of its financial applications—including fraud detection and credit scoring (Buchanan, 2019)—but not the exact techniques to apply. Getting from an idea to developing a model was partly remedied by hiring the ML specialist.

Although the quant risk team‐head possessed insufficient expertise to initiate the model development, he conjectured a probable solution (some kind of anomaly detection model) to manage the uncertainty of bank clients’ potentially abnormal trading behavior. Thus, the considerable transaction data made him think about new ways to mitigate counterparty risk. He knew that the uncertainty emanated predominantly from deliberate or unintended changes in the trading behavior in clients’ trading algorithms—mainly high‐frequency trading firms—and that the available transaction data might potentially absorb this uncertainty:

Clients are using more and more machines to do automatic trading, enabling them to send a few hundred orders per second. I do not believe algorithms or machines are perfect. A horrible scenario can be that algorithms will not function properly. It is something that we, at this moment, will not catch with our traditional statistical models […] In case the algorithms of a client are not functioning properly, if something is wrong, then I believe the new model—the trade‐anomaly‐detection algorithm—can identify patterns, which are different from the patterns made in the past by these clients. I can give you an example. If the normal trading pattern of a client is doing one trade a second or one trade every fifteen minutes and suddenly you receive ten thousand transactions in one second, that is suspicious, and I would want to know what is going on. The statistical models will not catch that. Though this relates more to operational risk than to market risk, I still want to know about patterns that differ from a client’s normal trading behaviour. In such a case, I will call the client and say, “Hey, is there something wrong?”, “Is everything okay on your side?”, “Did you change your trading strategy?” [K009, interviewee A]

Hence, the model transforms the uncertainty in client trading behavior into a more tangible form of risk. The model detects apparent anomalous trading behavior allowing on‐site risk managers, in daily contact with clients, to ask them whether the changed trading behavior reflects a deliberate strategic change or some algorithmic mishap. Some of the natural uncertainty surrounding firms’ trading behavior—the clearing bank does not know clients’ strategies, and clients do not know each other's strategies (at least, not in detail)—is therefore absorbed by the model. Specifically, the model converts noisy data into usable information through (1) identifying anomalous patterns in trades and (2) clarifying the cause of the anomaly by contacting clients. Moreover, the model does not merely absorb future uncertainty but operates at the junction of past, current, and future developments. It aims to detect how clients acted in the *past*, whether their *current* actions are consistent with past patterns, and what past and current behaviors may mean for *future* risk.

While the quant risk team devised a seemingly reliable ML model capable of absorbing natural uncertainty and improving risk management, the flipside of such complex models—especially unsupervised neural network ones—is the opacity from the scale of application in a specific domain (Burrell, 2016; Christin, 2020). The opacity and complexity of the model assemblages, combined with the flux and noisiness of financial markets, generate uncertainty in the model operations used to mitigate uncertainty. Thus, not only the model but its application to a complex system such as the financial market creates uncertainty‐inducing algorithmic opacity.

A senior director in one of the UK’s largest, most renowned quantitative hedge funds elaborated on the challenges of applying ML algorithms to investment management problems: “it is not like self‐driving cars or facial recognition software or something like that where there is something behind the data. In financial markets, there is a lot of spurious stuff behind the data. It changes very quickly” [K026]. Occasionally, the combination of noise, complexity, and opacity exposes doubt about the reliability and efficacy of the ML algorithm's operations, which we term *critical model uncertainty*. The following section examines how this uncertainty presents itself in practitioners’ reflections on their model use.

## 5 MULTIPLICATION OF CRITICAL MODEL UNCERTAINTY

“To me, it looked very random, I mean, to the point where I got very sceptical of anybody that tells me they use machine learning for trading” [D021]. These words of a software developer, previously employed in a Chicago high‐frequency trading firm but transitioned to a technology firm, reflect his experience with people hired by his old trading firm to recreate ML supported trading strategies that had made money elsewhere. It seldom worked as intended. The developer likened the working of the ML models in the trading space to a coin toss saying the randomness was due to users and developers being unsure about what their machines were doing. This kind of skepticism toward ML is not rare in the industry. The skepticism derives from opposition to change and/or distrust in the algorithms’ ability to “learn” and optimize without human intervention and intermediation (Hansen, 2020a).

The concerns many practitioners have about ML models in finance settings relate to comprehensibility and control. If some operations are incomprehensible, the resultant inexplicability breeds uncertainty. This is not a big problem if the model's logic is interpretable, but it is if those developing and deploying the models cannot explain how their models arrive at a given output prediction. This applies to clearing operations as detailed and firms that apply ML models to generate trading strategies (we interviewed several firms specializing exclusively in ML‐based trading). As a portfolio manager‐turned‐analytics‐provider explained, asset management firms, in particular, might not want an overly opaque model: “they will need to understand how you got to that answer.” He argued that developers and users need to balance transparency and complexity. The model ought to be sufficiently transparent to exhibit its basic logic and complex enough for its application context and to avoid it being easily copied [K038] (Hansen, 2020b). To illustrate the problem, global asset management leaders such as the MAN Group and BlackRock shelved ML models they found too opaque and thus impossible to explain (Kilburn, 2018; Satariano & Kumar, 2017).

Black box models can be hard to abandon if they perform consistently well in backtests, but it is fear of not knowing what is going on if the model starts “misbehaving” that makes such models unappealing to many in the financial industry. A quant devising trading models in a large UK‐based hedge fund touched upon the balance (in this case, tension) between model performance and lack of transparency:

We did, at some point, use one model, which was actually doing fantastically well, but we could not get it past senior management for exactly that reason. “It is too much of a black box model”, senior management said. “You cannot explain where the predictions are coming from, so we are not going to trade on it, even if it has done well in backtests for ten years”. […] In some sense, it was a shame. It was the best we had ever done. But, without being able to explain where the money is coming from, we cannot trade it. [K031]

Here, it was impossible for developers to explain the model's functioning. Parts of what was happening “under the hood” of the model were essentially incomprehensible to them. In line with this, our interview data suggest that, except a smaller number of firms that are entirely committed to complex ML models, traders and portfolio managers tend to be uncomfortable with not knowing or having a hard time understanding the operations of certain complex ML trading models. Many traders tend to be reluctant to allocate money to algorithmic strategies that they cannot fully comprehend. However, as a quant working in a USA‐based hedge fund explained, the same uneasiness exists with more technically apt quants:

If the trader or portfolio manager does not understand the model, he would never put money on it. You are limited by the skillset in the industry. […] To be fair, many traders these days come with math or physics backgrounds, but if you do not practice for a couple of years you forget it all, and then you cannot understand it anymore. They would never put money on something they do not understand and if it is something recurrent that they experienced over the years, you would have to be able to explain things, otherwise it is dead. That is why they tend to use simple models. From a quant point of view, it makes total sense too. I remember when we were pricing exotic products. Some products were so complicated, it was very difficult to understand the risk implications in such or such market environment. If you are not at ease with a model and what it can do when it is stressed this or that way, then it is very risky. […] This is why linear regression models are always going to be around, because they are intuitive. Neural nets are not at all. They are intuitive in what they are doing, but if you start having like ten or more layers, you cannot keep track. [K039]

The basic yet significant point here is that it is necessary to know the fundamental logic of a model (how it gets to its output). Otherwise, comprehensive risk assessments are jeopardized; if the model is not “intuitively” intelligible, in the hedge fund quant's words, it becomes challenging to foresee its reaction to different “stress” events, such as rare, high‐impact “tail events” (Barberis, 2013) like the 2008 global financial crisis or the COVID‐19 pandemic.

Moreover, if the pattern‐extraction logic of the model is not known, it may be more difficult for its designers and users to assess and detect whether the model is “overfitted.” In simple terms, a model is said to be overfitted if it corresponds too closely or exactly to the features of the data set on which it has been trained. Overfitting is a rather mundane yet highly important problem in ML modelling and it often refers to a situation in which a model acts on idiosyncratic noise as if it were information and, consequently, identifies patterns and correlations in the data that might be spurious. The greater the complexity of the data set, the higher is the likelihood of the model overfitting on noise (Hansen, 2020b, pp. 6–9). Overfitting is most often detected as a performance discrepancy between *in‐sample* and *out‐of‐sample* testing of a model. The in‐sample testing is the learning phase where the model runs of training data, whereas the out‐of‐sample testing is done on data not used in the training phase. If the model performs wonders in‐sample, but fare horribly out‐of‐sample, it is a clear indication of overfitting (Varian, 2014, pp. 7–8).

The risk of models fitting on noise and, as a result, potentially leading them astray with predictions based on spurious correlations was a concern to several of our informants. To be specific, 12 of our 45 informants working with ML models discussed overfitting, seeing it as an imminent problem and a source of risk they tried to alleviate. To this end, some would seek to constrain model complexity by striving toward parsimony and adhering to Occam's Razor as a rule of thumb in modeling (Hansen, 2020b). Although heuristics like Occam's Razor can serve as guiding principle in some contexts, it does not do away with the inherent opacity of more complex models such as deep neural networks. Given this, the concern with overfitting is a main reason why many of the quants and traders we have talked to prefer more “intuitive” models or at least try to constrain model complexity and thus, model incomprehensibility.

We have argued that the opacity of some ML models introduces critical model uncertainty, which can make it difficult to detect whether a model is overfitted. Although ML models absorb natural market uncertainties and transform these into manageable risk, how neural network‐based models do so remains unexplainable for most market participants. The critical uncertainty issue with these models is not (merely) that their action can be subjected to varying interpretations that conjure up incommensurable rationales, but rather that these models tend to evade interpretable rationales altogether.

One implication of critical model uncertainty is that organizational power assumes a new role in organizations that pursue sophisticated ML. According to March and Simon (1993, p. 187), “uncertainty absorption is frequently used, consciously or unconsciously, as a technique for acquiring and exercising power.” Specifically, those in charge of uncertainty absorption will often define the premises of organizational action and decision‐making. While March and Simon's theorization refers to the capacity of humans to exercise power through uncertainty absorption, the rise of ML and its critical model uncertainty suggest that the loci of such power increasingly reside in machines. However, similar to how we do not consider Knight's distinction between risk and uncertainty as historically invariant, we do not argue that critical model uncertainty cannot be, at least, partially alleviated. Computer scientists are conscientiously developing methods to better explain artificial neural networks (Samek et al., 2019). Through these attempts, possibly, humans will be able to explain how and why ML models arrive at their output predictions. This would alleviate some of the critical uncertainty we attribute to ML models and rebalance the power relations between humans and machines.

## 6 CONCLUSION

The widespread experimentation with and use of ML techniques in the financial industry signal an imminent paradigm shift in trading and investment management (Lopez de Prado, 2018). These advanced statistical tools unwrap possibilities to process masses of market data and alternative data sources, occasionally discovering opportunities (data patterns, correlations, anomalies) that no human analyst, trader, or portfolio manager would have identified. These techniques challenge and shape financial practitioners’ perceptions of what constitutes relevant data, whether it is wise to trust human judgment, to what extent model operations ought to be fully transparent, etc. Consequently, some ideas that previously explained financial markets and their guiding principles are renegotiated, bypassed, or subverted as more automated machine‐ and data‐driven approaches to investment management, trading, and risk management infiltrate the industry.

With the gradual move toward increasing automation and using learning algorithms, this article has explored what becomes of uncertainty and how this intangible phenomenon is rendered palpable and emerges in new ways as trading and investment and risk management systems are constructed around adaptive, big‐data sifting ML techniques. Some ML techniques blur the distinction between quantifiable risk and unpredictable uncertainty by transforming what the boundedly rational human only perceives as impalpable uncertainty into quantifiable, manageable risk. Thus, certain ML techniques promise to render uncertain futures probable (subject to statistical analysis). Moreover, with an explosion of data availability in finance and elsewhere, uncertainty is no longer reserved for the future. Increasingly, immense market activity renders the past and the present uncertain, as it is challenging to establish what happened even in the immediate past and how it might affect the immediate future. ML systems are deployed to deal with these uncertainties as well.

While we show that ML techniques help transform past, present, and future uncertainty into risk, we argue that the application of ML in finance is significant for how financial practitioners perceive and manage uncertainty. Our fieldwork in the trading and investment management industries suggests that while ML models help absorb natural uncertainties, other uncertainties emerge from these highly complex, adaptive, and opaque models in ephemeral contexts like financial markets. This leads to what we call critical model uncertainty—the inability to understand the inner workings of those models that absorb the natural uncertainties of markets.

We suggest that critical model uncertainty and its dialectical relation to ML systems’ absorption of natural uncertainty need further study, including in non‐financial contexts. Not only does critical model uncertainty reshuffle power configurations in organizations, but it highlights the core problem with ML systems—that humans remain largely ignorant about the ways in which these systems’ uncertainty‐absorbing accomplishments are achieved.

## CONFLICT OF INTEREST

There is no conflict of interest.

## ETHICAL APPROVAL

Research for this article follows the ethical guidelines of the BSA and ASA.

## Data Availability

Research data are not shared. The reason is that informants have been promised full anonymity.

## ENDNOTES

^1^
Christin (2020) outlines three approaches to the ethnographic study of algorithms. The approach dubbed “algorithmic refraction” most closely resembles our approach to studying ML model use in financial markets. The concept of refractions, Christin indicates, is derived from physics, “where it refers to the changes in direction and strength that occur whenever a wave of light or sound passes from one medium to the next” (2020, p. 906). Applied to the ethnographic study of algorithms, refraction means treating algorithms as “prisms that both reflect and reconfigure social dynamics” that enable the study of “complex chains of human and non‐human interventions that together make up algorithmic systems” (p. 907).

^2^
In addition to the authors, Daniel Souleles, Bo Hee Min, Nicholas Skar‐Gislinge, and Pankaj Kumar contributed to the data collection.

^3^
The bank's quant risk team at the time of the interview comprised ten people. Apart from the ML specialist, most team members were financial economists. The team‐leader had studied mathematics, finance, and computer science. In addition to the team of quants in the bank's offices in Europe, several on‐site risk managers monitor clients’ risks locally and report back to the main office. Other than the interview, the first author participated in an industry conference where the ML specialist and another quant risk team member presented their anomaly detection model.

## 1

**TABLE A1:** Total interviewees

Type	Number
Trading firms (High‐frequency trading firms in particular)	61
Asset management firms	12
Hedge fund management firms	19
Banks	19
Broker, broker‐dealer firms	10
Exchanges and other trading venues	23
Regulators	10
Data, technology, and analytics vendors	15
Other	13
Total	182

**TABLE A2:** List of interviews in machine learning subsample

Interview ID	Type of firm	Role	Location	Date
C002	Investment bank	Developer	London	November 6, 2017
C005	Hedge fund	Machine learning researcher	London	November 7, 2017
C006	Algorithmic trading firm	Machine learning engineer	London	November 22, 2017
D006	Hedge fund	Sr. research scientist	New York	December 12, 2017
D012	Hedge fund	Head of computer trading	New York	December 6, 2017
D020	Algorithmic trading firm	Trading operations specialist	Chicago	September 27, 2017
D021	Algorithmic trading firm	Software developer	Chicago	September 26, 2017
D024	Hedge fund	Trading algorithm engineer	Chicago	October 25, 2017
D029	Algorithmic trading firm	Algorithmic trading lead	Chicago	October 20, 2017
D032	Algorithmic trading firm	Fund manager	Chicago	October 16, 2017
D033	Hedge fund	Chief scientist and CTO	San Francisco	January 22, 2018
D038	Algorithmic trading firm	Quantitative trading analyst	Chicago	January 24, 2018
BC001	Algorithmic trading firm	Founder and CEO (two persons)	London	August 30, 2018
BC003	Algorithmic trading firm	Sr. Software Engineer	London	August 30, 2018
BC004	Algorithmic trading firm	Delivery managers (two persons)	London	August 30, 2018
BC005	Algorithmic trading firm	Head of market risk	London	August 31, 2018
BC006	Algorithmic trading firm	Delivery manager, software engineer, and compliance officer (three persons)	London	August 31, 2018
BC007	Algorithmic trading firm	CTO	London	August 31, 2018 and November 28, 2018
BC008	Algorithmic trading firm	Infrastructure engineer	London	August 31, 2018
BC009	Algorithmic trading firm	CEO	London	February 28, 2019
BC010	Algorithmic trading firm	CEO and CTO (two persons)	London	February 28, 2019
BC011	Algorithmic trading firm	Production team	London	March 1, 2019
BC012	Algorithmic trading firm	Leadership team	London	March 1, 2019
BC015	Algorithmic trading firm	CRO	London	August 29, 2019
BC016	Algorithmic trading firm	Trader	London	August 29, 2019
BC017	Algorithmic trading firm	CEO and CTO (same two persons as BC010)	London	August 29, 2019
K007	Pension fund	Quantitative portfolio manager	London	January 30, 2018
K009	Clearing bank	Head of quant risk team and machine learning quant (two persons)	Amsterdam	April 12, 2018
K012	Analytics vendor	Machine learning quant	New York	May 29, 2018
K013	Consultant	Quant trader and machine learning specialist	Spain	May 31, 2018
K017	Hedge fund	Quant analyst	Paris	June 19, 2018
K018	Hedge fund	Researcher	London	June 25, 2018
K019	Analytics vendor	Head of research	London	June 26, 2018
K024	Brokerage firm	Global head of product management and head of EMEA	London	September 5, 2018
K026	Hedge fund	Director of investment strategies	London	September 6, 2018
K027	Technology vendor	CSO	London	September 20, 2018
K029	Hedge fund	Deputy head of research	London	October 11, 2018
K031	Hedge fund	Quantitative researcher	London	November 2, 2018
K038	Analytics vendor	CEO	London	November 22, 2018
K039	Hedge fund	Senior quantitative analyst	London	December 20, 2018
K040	Hedge fund	Fund manager	London	March 5, 2019
K041	Investment bank	E‐trading risk quant	London	March 28, 2019
BK001	Brokerage firm	Quantitative researcher and machine learning quant (two persons)	London	September 26, 2018
BK002	Brokerage firm	Head of quantitative trading	London	June 6, 2019
G002	Algorithmic trading firm	Algorithmic trading lead	Chicago	May 23, 2018
